# An experimental study of the optimum position of the drainage gallery beneath concrete gravity dam

**DOI:** 10.1038/s41598-026-65760-6

**Published:** 2026-08-22

**Authors:** Reham Salem, Mohamed Abd El-Razek, Magdy M. Aboelela

**Affiliations:** Irrigation Engineering and Hydraulics Department, Faculty of Engineering, Alexandria University, Alexandria, 11432 Egypt

**Keywords:** Gravity dams, Uplift pressure, Drainage gallery, Hele-Shaw model, Seepage discharge, Energy science and technology, Engineering, Hydrology

## Abstract

A gravity dam relies primarily on its self-weight for stability, making the control of uplift pressure beneath the foundation essential. This study experimentally investigates the optimal horizontal and vertical placement of a drainage gallery using a Hele–Shaw model constructed in the Irrigation and Hydraulics Department laboratory at Alexandria University. The relative horizontal position of the gallery is expressed as (x/B), where (x) is the distance from the dam heel and (B) is the base width, while the relative depth is expressed as (d/H), where d is the gallery depth and (H) is the upstream water height. The investigation assumes a steady-state seepage condition through a simplified, homogeneous, and isotropic foundation material beneath the dam. The values of parameter (x/B) in the experiment are 0.11, 0.28, 0.44, 0.61, and 0.78 respectively and the values of parameter (d/H) are 0.026, 0.061, 0.096, 0.130, and 0.165 respectively. Fluid viscosity and hydraulic conductivity were dynamically calculated based on oil temperature variations. Results show that the most effective configuration which minimize the uplift pressure (maximum reduction in uplift pressure force) and maximize the seepage quantity passing through the drainage gallery occurs at (x/B) = 0.15 and (d/H) = 0.165, achieving nearly 70% reduction in uplift pressure. Also, increasing the depth of the drainage gallery (d/H) increases the seepage quantity and decreases the uplift pressure force. In comparison, the theoretical formulation of^11^ indicates an optimum at (x/B) = 0.125, with a predicted reduction of approximately 56%. Additionally, increasing the horizontal distance of the gallery reduces seepage discharge and increases the uplift pressure. These experimental findings offer refined geometric guidelines for gravity dam foundation design, allowing engineers to enhance structural safety margins against sliding, optimize foundation drainage layouts, and reduce excessive conservative safety factors in hydraulic infrastructure projects. Also, these findings provide preliminary experimental guidance that should be validated for different geometries and site conditions.

**Supplementary Information:**

The online version contains supplementary material available at 10.1038/s41598-026-65760-6.

## Introduction

The concrete dam is essential because it provides many benefits. Such dams supply reliable, long-lasting water for irrigation, hydropower, flood control, and drinking water. They are crucial for water needs, regulating river flow, and supporting economic growth in many areas. However, seepage through the dam foundation continues to be one of the most challenging stability concerns (Chen et al., 2016; Norouzi Sarkarabad et al., 2017)^1,2^. Therefore, it is crucial to implement proper seepage control measures and monitoring systems to ensure the safety and long-term stability of concrete dams (Clarkson and Williams, 2021)^3^.

Water pressure causes seepage under dams through permeability zones such as cracks and joints. The Vyrnwy dam, built between 1882 and 1891 in Wales, was the first to recognize the significance of dam-foundation permeability and related uplift stresses^4^. Seepage through the dam body and foundation can reduce the stability of concrete and masonry dams. It may cause material leaching, internal erosion, and increased uplift pressure, as highlighted by the failure of the Bouzey Dam in France^5^.

Seepage impacts on hydraulic structure foundations can be divided into three types: uplift force, seepage discharge, and existing gradient. The uplift force diminishes the shear strength between the structure and its foundation, which can compromise the dam’s stability by increasing the risk of slipping or overturning^6^. Strong uplift forces can cause water to seep through the concrete surface, allowing water and harmful substances, such as waterborne contaminants and chemicals, to enter the dam^7^. Furthermore, excessive uplift pressures beneath a concrete dam can significantly threaten its structural integrity. Uplift decreases the dam’s effective weight, making it more vulnerable to slipping or overturning and possibly leading to deformations like uneven uplift, tilting, or bending, which in severe cases can result in failure^8,9^. Therefore, effective drainage systems are essential for dissipating pore pressures and enhancing safety.

Research on seepage control beneath gravity dams spans analytical, numerical, and experimental approaches. Analytical studies have provided fundamental insights into seepage and uplift behavior. For instance, Chawla et al.^10^, derived ideal drain positions using seepage theory, while USBR formulation^11^ offers widely used analytical relations for estimating uplift and seepage under idealized conditions. These methods are valuable for preliminary design but assume simplified conditions and cannot capture complex interactions between multiple drainage parameters.

Numerical investigations have explored drainage design parameters in greater detail. Utili et al.^12^, applied equilibrium and distinct element methods to assess uplift pressure and reservoir level effects with and without drainage galleries. Aghdam et al.^13^, and Nourani et al.^14^, used SEEP/W and FEM models to study the influence of drain diameter and position on uplift pressure, exit gradient, and discharge. Gao et al.^15^, examined the effects of drainage gallery position on dam stresses using ANSYS, while Da Silva^16^, conducted a comprehensive 3-D FEM analysis varying drain position, number, diameter, spacing, and roughness. Optimization-focused studies include^17^, who employed CADAM to identify ideal drainage gallery position and drain diameter, and^18^, who compared drains and sheet piles beneath gravity dams using FEM. Recent work by^19^ provides valuable numerical insight into the horizontal placement of drains. Their simulations show a distinct minimum in uplift reduction. Importantly, this minimum is broad and shallow, spanning a wide range of relative distances (approximately 0.1L–0.35L), rather than forming a sharp optimum. This behavior indicates that multiple drainage positions yield nearly identical uplift reductions, making it difficult to specify an exact optimum based solely on numerical analysis. Furthermore, a lot of these numerical studies primarily addressed horizontal positioning or diameter in isolation, leaving the combined effect of horizontal and vertical placement underexplored.

Several experimental models, such as Hele-Shaw and sand-tank models, have been employed in earlier research to examine the performance of drainage galleries. Sand-tank models can replicate soil seepage, but they need to be carefully prepared to create consistent conditions. Hele-Shaw models are simpler and enable precise measurement of 2-D seepage flow in controlled laboratory settings. They are helpful for comparing the hydraulic performance of various seepage-control systems, even if they cannot fully capture the characteristics of actual soil. For this reason, the Hele–Shaw model was selected in the present study to evaluate the effect of different drainage gallery configurations.

Experimental studies have demonstrated the practical influence of drainage systems, but remain limited in scope. Khalili Shayan and Amiri-Tokaldany^20^ showed that the presence and placement of drains significantly reduce pore pressure. Abd El-Razek and Elela^21,22^ used sand models to examine drainage gallery positioning under varying upstream heads and drain diameters. Despite these efforts, experimental investigations have not systematically varied both horizontal and vertical coordinates, nor have they visualized seepage patterns in detail. Under stable, laminar, and two-dimensional conditions, the Hele-Shaw model is appropriate for assessing the relative effects of seepage-control techniques, such as blanket length, cutoff depth, and foundation thickness of earth dams^23^.

Across analytical, numerical, and experimental domains, the optimal configuration for drainage galleries, whether horizontal or vertical, remains insufficiently defined. Most studies focus on single parameters or rely on simulation assumptions, while controlled physical experiments addressing combined positioning are unexplored. This study addresses existing gaps by employing a controlled Hele-Shaw analog modeling approach. To address these gaps, the controlled Hele-Shaw analog modeling approach is used in the present study. The Hele-Shaw model is particularly suitable due to its ability to accurately simulate two-dimensional potential flow fields and effectively visualize seepage patterns^24^. A systematic experimental investigation was conducted to determine the optimal horizontal and vertical placement of drainage galleries beneath dams. In addition, we use the formulation of Eq. 1 in the theoretical section as a benchmark for comparing experimental uplift-reduction predictions^11^. USBR textbook offers analytical relations widely used in practice to estimate seepage and uplift behavior. Therefore, it serves as an appropriate theoretical reference for validating and contextualizing experimental results. The main objective of the study is to investigate the influence of the drainage gallery position on the uplift pressure force reduction and the seepage discharge using the experimental model (Hele-Shaw model).

## Experimental set-up

### Hele-Shaw model

**Fig. 1:**
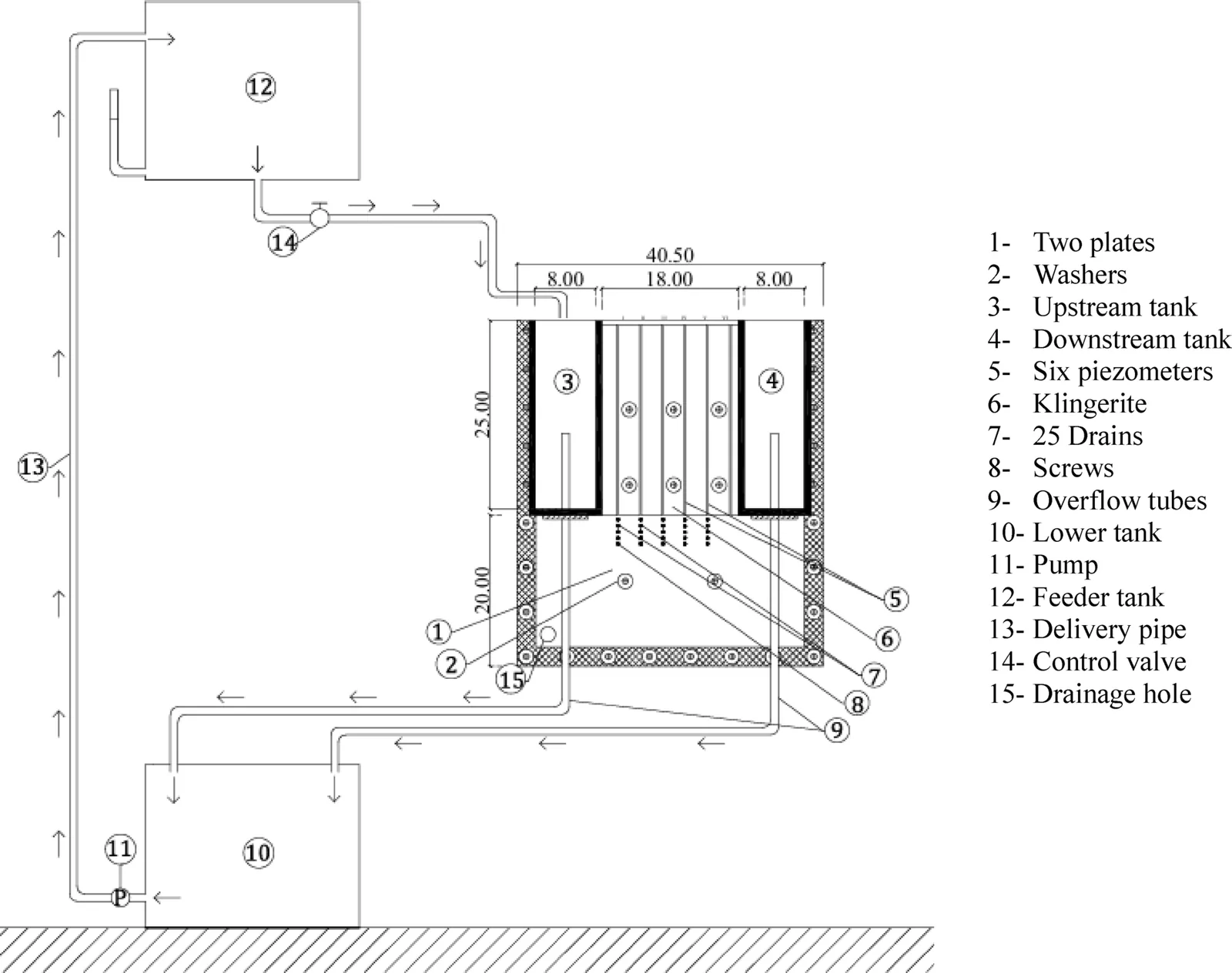
Hele-Shaw model (Dimensions in cm).

**Fig. 2:**
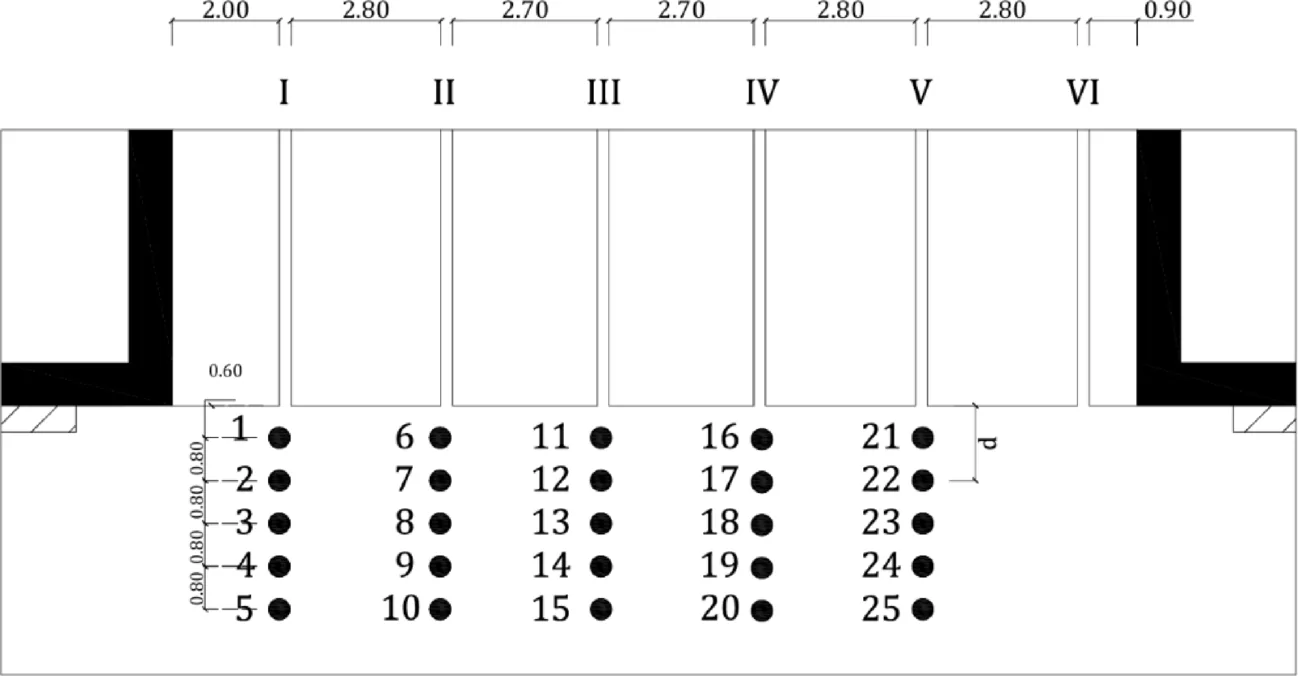
Drainage gallery distances and depths.

The Hele-Shaw model is designed to represent flow through gravity dams (Fig. 1). The model consists of two Perspex plates (1). The 1.86 mm distance between the two plates is maintained by washers (2). The model has an upstream tank (3) to create the retained head upstream of the dam. Another tank (4) is located downstream to achieve tailwater or dry downstream of the dam. The model contains six piezometers (5) manufactured from Klingerite material (6) to measure the uplift pressure. Twenty-five holes (7) in the dam floor are constructed to simulate drainage galleries (Fig. 2). A screw (8) controls the exit seepage discharge from the gallery. Overflow tubes (9) are used to change upstream and downstream heads. The excess oil (7500 20W/B) from tanks (3) and (4) is directed to a lower tank (10), which is pumped (11) to the feeder tank (12) through a pipe (13). A valve (14) is used to control the discharge quantity of the oil feeding from the tank (12). A lower hole (15) is made to empty the model of oil. A thermometer is immersed in the oil of the upstream tank to measure its temperature.

### Methodology

**Fig. 3:**
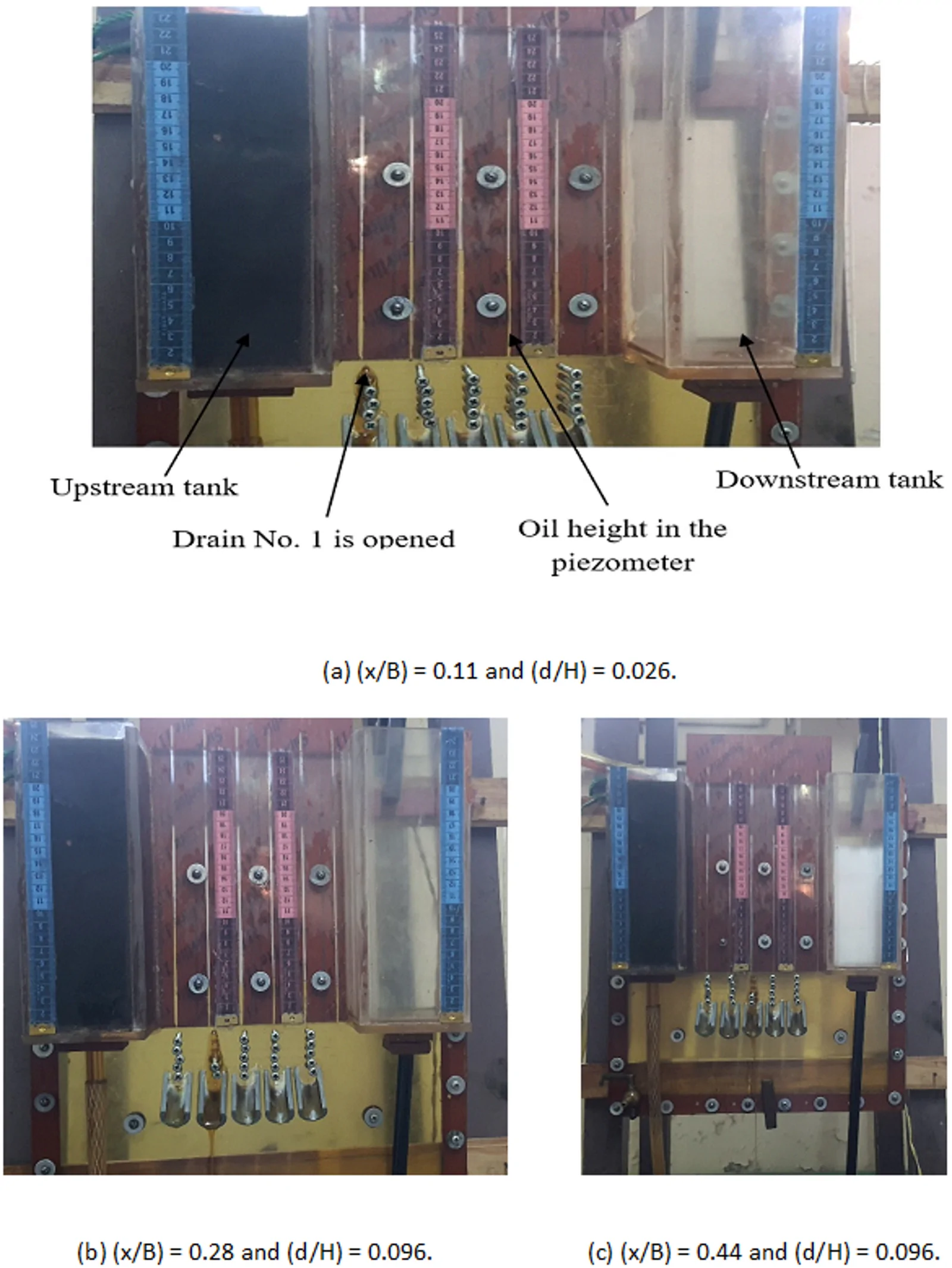

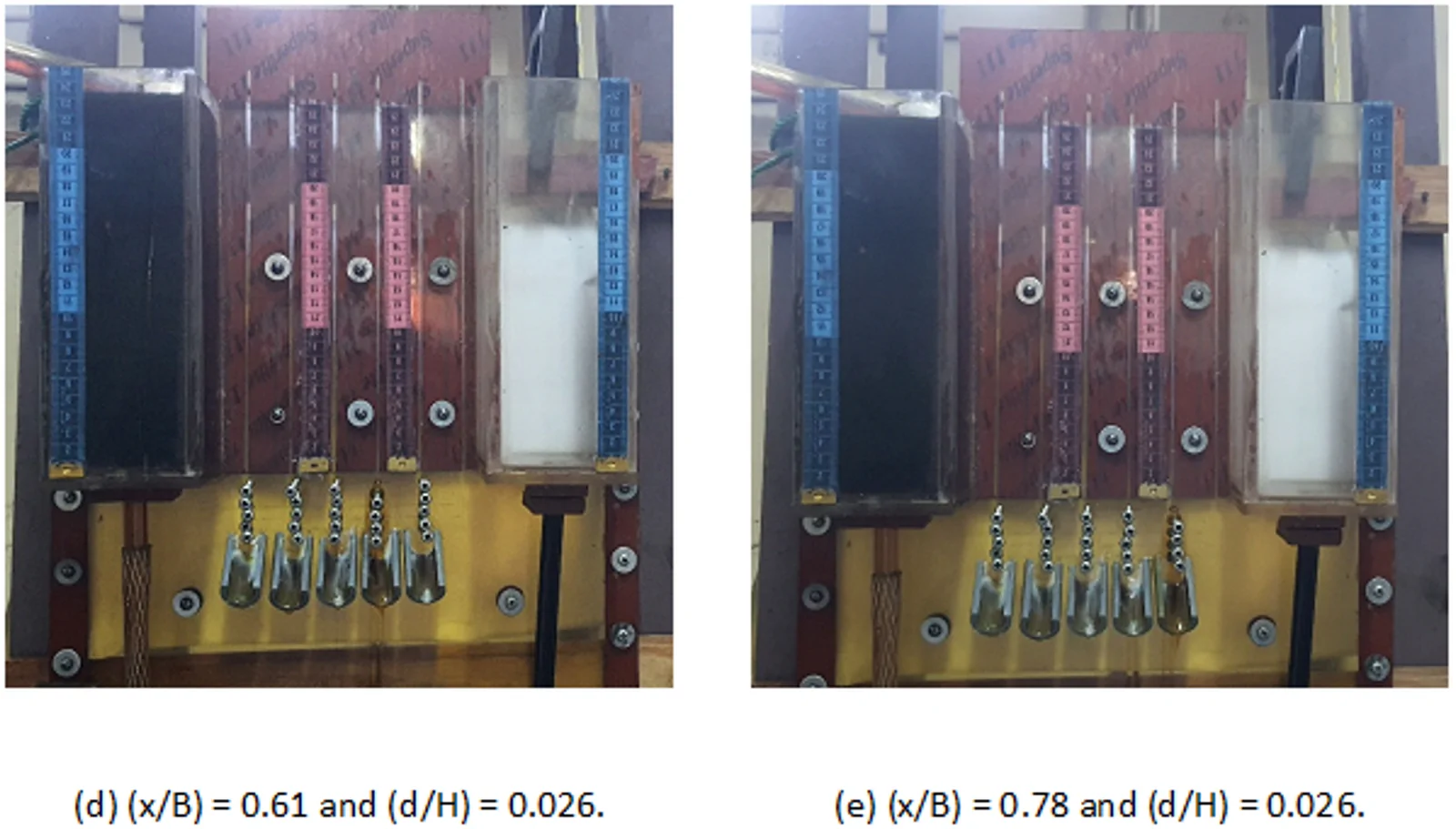
(**a**–**e**) Hele-Shaw model during experiment for different (x/B) and (d/H).

The drainage gallery was simulated using a series of pre-drilled holes within the dam foundation model, aligned at the same elevation to represent a horizontal drainage tunnel. The depth ratio (d/H) was controlled by selectively activating different sets of these holes located at various depths, where each set corresponds to a specific gallery level and was tested independently. The flow within the Hele–Shaw model was maintained under laminar conditions, as confirmed by the low Reynolds number. According to Hele–Shaw theory, the system behaves as an equivalent porous medium where permeability is governed by the gap width and fluid viscosity. The experiments were conducted under monitored laboratory temperature conditions, and the corresponding oil viscosity was determined for each test based on the measured temperature. Thus, variations in viscosity due to temperature changes were explicitly accounted for in the analysis. Figure 3a–e represents some photographs of the Hele-Shaw model that were carried out in the laboratory. Figure 3a, represents experiment for (x/B) = 0.11 and (d/H) = 0.026 (drain No. 1 is opened) and then piezometers reading and seepage quantity of the oil are measured. Figure 3b represents experiment for (x/B) = 0.28 and (d/H) = 0.096 (drains No. 6, 7, and 8 are opened). Figure 3c represents experiment for (x/B) = 0.44 and (d/H) = 0.096 (drains No. 11, 12, and 13 are opened). Figure 3d represents experiment for (x/B) = 0.61 and (d/H) = 0.026 (drain No. 16 is opened). Figure 3e represents experiment for (x/B) = 0.78 and (d/H) = 0.026 (drain No. 21 is opened). Also, it is clear from Fig. 3a that the upstream tank contains oil while the downstream tank is empty of oil (dry downstream case).

## Results and discussion

### Seepage discharge analysis:

**Fig. 4:**
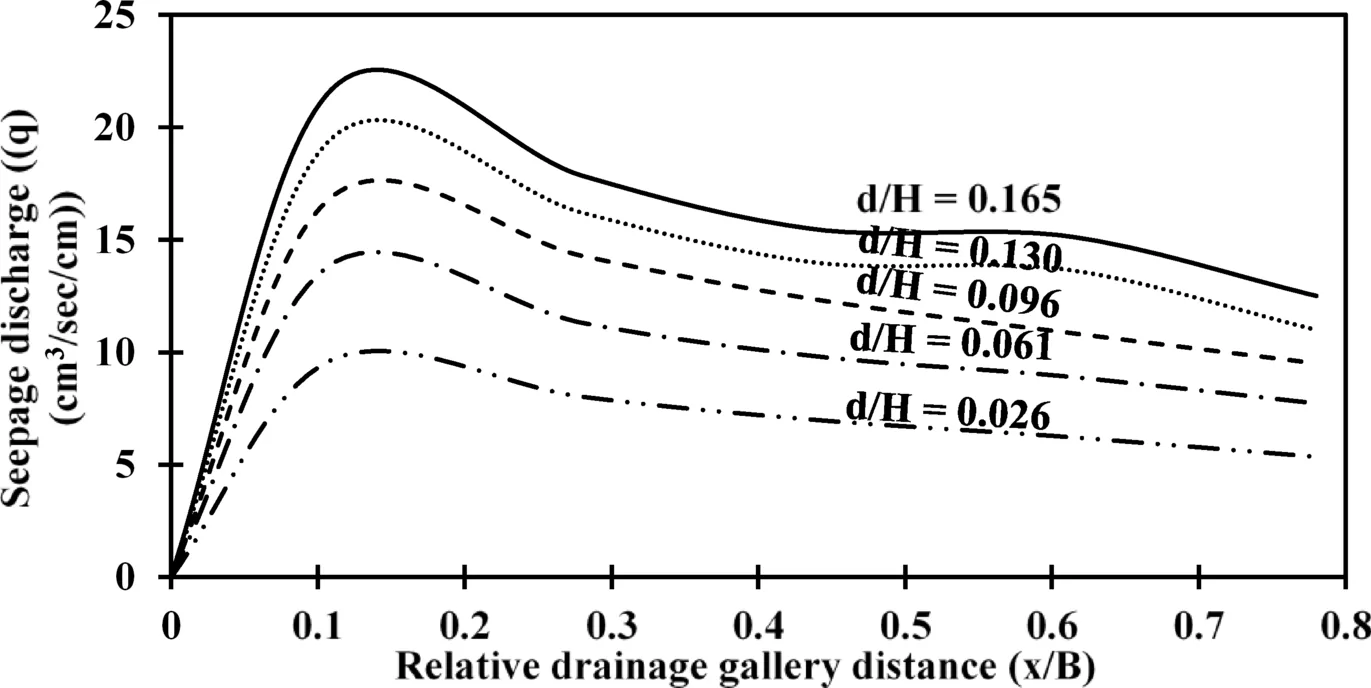
Seepage discharge (q, cm^3^/sec/cm) versus relative drainage gallery distance (x/B).

Table 1 and Fig. 4 show that the maximum seepage discharge occurs at a relative drainage gallery distance (x/B) equal to 0.15 measured from the heel. (x/B) = 0 corresponds to a dam without a drainage gallery. The seepage discharge increases with increasing drainage gallery depth and decreasing distance from the heel. The present results indicate an optimal drainage gallery location at (x/B) ≈ 0.15 and (d/H) ≈ 0.165, achieving nearly 70% reduction in uplift pressure. This differs from Abd El-Razek and Elela^21^, who reported an optimal position at (x/B) ≈ 0.5 using a sand model, and observed a weaker effect of depth. These discrepancies can be attributed to differences in model type, boundary conditions, and assumptions. The Hele-Shaw model utilizes a highly viscous fluid flowing through a micro-gap between two parallel transparent plates, mathematically replicating two-dimensional planar potential flow through porous media according to Darcy’s Law and the Laplace equation. The key advantage of this narrow-gap physical model is its exceptional visual clarity: by injecting colored dyes, streamline flow nets can be observed in real time beneath the dam foundation. This allows for precise, direct tracking of seepage paths and clear visualization of how horizontal and vertical drainage gallery positioning alters flow patterns and mitigates uplift pressure. The Hele-Shaw model used in the present study simulates 2-D flow with clear visualization of seepage paths, while sand models may exhibit scale-dependent seepage behavior. Additionally, differences in tailwater levels and dam geometry can influence the relative effect of gallery placement on uplift reduction.

**Table 1:** The seepage discharge (q, cm^3^/sec/cm) at different relative drainage gallery distances (x/B) and depths (d/H).

(x/B)	0	0.11	0.28	0.44	0.61	0.78
(d/H)	q (cm^3^/sec/cm)					
0.026	0	9.67	8.02	6.99	6.23	5.35
0.061	0	13.95	11.31	9.82	8.93	7.73
0.096	0	16.93	14.28	12.33	10.88	9.51
0.130	0	19.56	16.21	13.99	13.68	11.00
0.165	0	21.73	17.85	15.46	15.17	12.51

### Uplift pressure force analysis due to drainage gallery position:

**Fig. 5:**
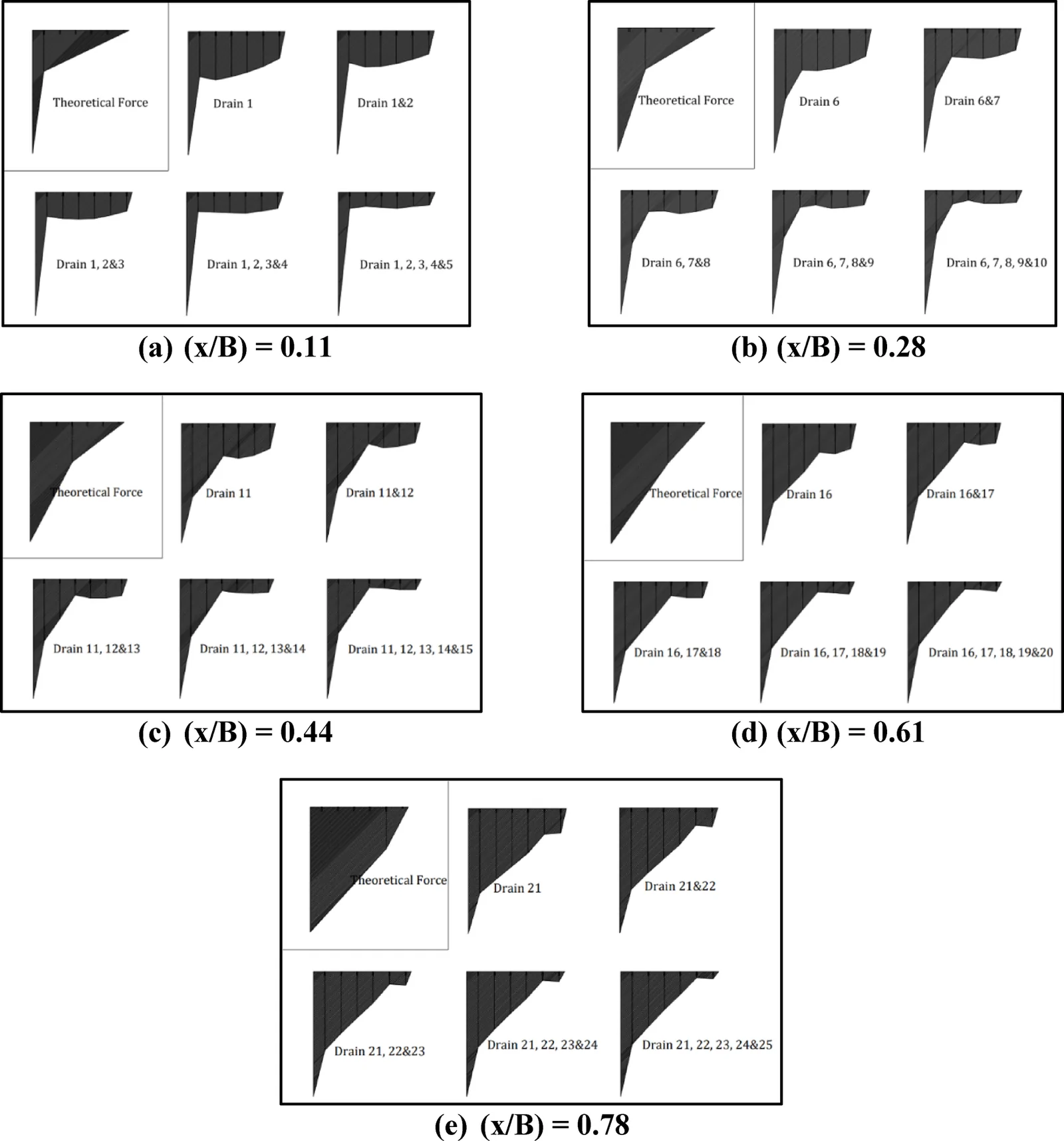
(**a**–**e**) Uplift pressure diagrams measured from experimental and theoretical cases.

The uplift pressure force beneath a gravity dam with a drainage gallery can be theoretically calculated using Eq. 1^11^,. This is to compare its value (U) with that produced from experimental work. Also, for a gravity dam without a drainage gallery, this force (U_o_) is experimentally and theoretically calculated to determine the percentage reduction in the uplift force (U.R.) caused by the drainage gallery, as listed in Table 2. Figure 5a–e indicates uplift forces in the case of theoretical and experimental work for (x/B) = 0.11, 0.28, 0.44, 0.61, and 0.78.

1 $$\mathrm{p}={\upgamma}_{\mathrm{w}}{\mathrm{H}}_{\mathrm{D}\mathrm{S}}+\frac{1}{3}\left({\upgamma}_{\mathrm{w}}\mathrm{H}-{\upgamma}_{\mathrm{w}}{\mathrm{H}}_{\mathrm{D}\mathrm{S}}\right).$$

where: p is the pressure at the drainage gallery position (t/m^2^), H_DS_ is the downstream water height (m), and H is the upstream water height (m).

**Table 2:** Relative measured uplift pressure force (U/U_o_) and uplift pressure force reduction (U.R.) at different relative drainage gallery distances (x/B).

(x/B)	0	0.11	0.28	0.44	0.61	0.78	0	0.11	0.28	0.44	0.61	0.78
(d/H)	(U/U_o_)						(U.R.)					
0.026	1	0.65	0.67	0.69	0.73	0.79	0	0.35	0.33	0.31	0.27	0.21
0.061	1	0.53	0.54	0.57	0.63	0.71	0	0.47	0.46	0.43	0.37	0.29
0.096	1	0.43	0.45	0.50	0.56	0.65	0	0.57	0.55	0.50	0.44	0.35
0.130	1	0.37	0.39	0.44	0.50	0.60	0	0.63	0.61	0.56	0.50	0.40
0.165	1	0.30	0.33	0.39	0.46	0.56	0	0.70	0.67	0.61	0.54	0.44

It is clear from the diagrams that the minimum uplift pressure occurs when the drainage gallery is positioned nearest the upstream side. Additionally, as the depth of the drainage gallery increases, the uplift pressure decreases. The drainage gallery depth has no effect on the theoretical distribution, because it is not considered in the USBR equation.

**Fig. 6:**
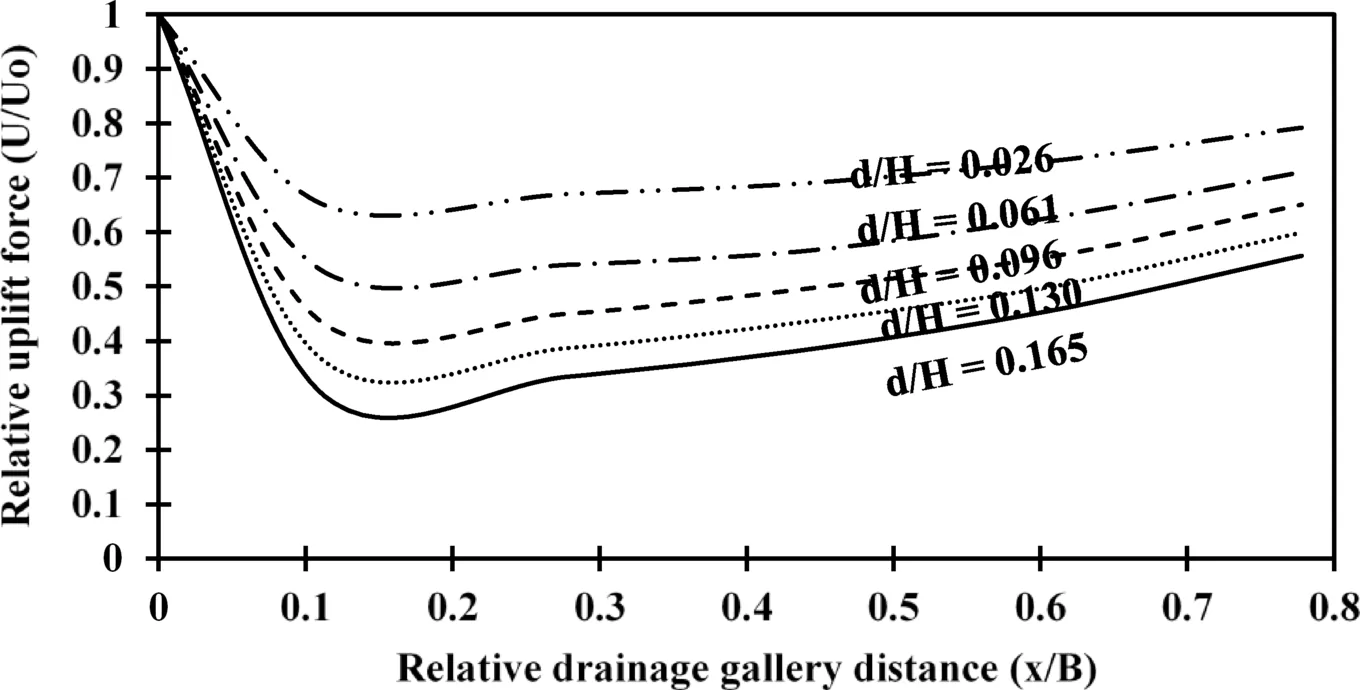
Relative uplift force (U/U_o_) versus relative drainage gallery distance (x/B).

**Fig. 7:**
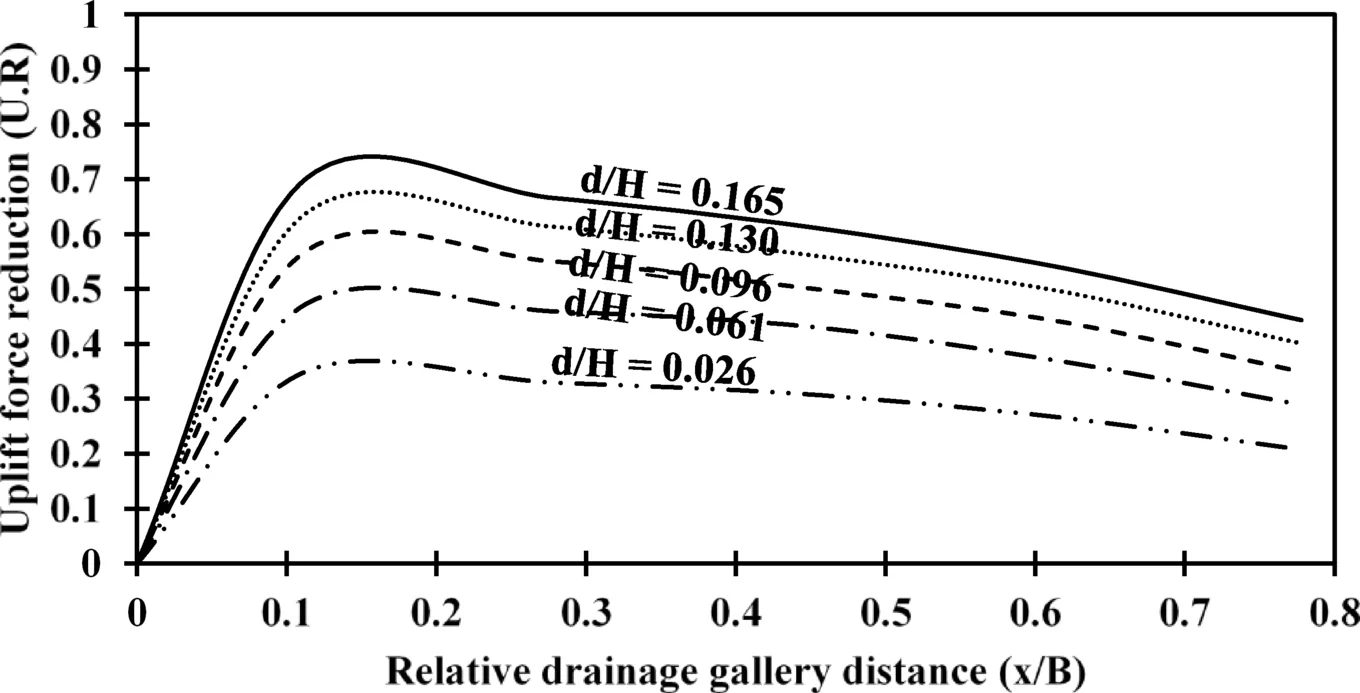
Uplift pressure force reduction (U.R.) versus relative drainage gallery distance (x/B).

Figure 6 shows the relation between the relative drainage gallery distance (x/B) versus the relative uplift force (U/U_o_). From the figure, it is evident that the uplift force decreases with increasing depth of the drainage gallery (d/H). Also, the minimum value of the uplift force at a distance (x/B) from the upstream face of the dam is 0.15. The reduction in this force (U.R.) reaches 70% for (d/H) = 0.165, as shown in Fig. 7. The optimal drainage gallery location and the observed uplift reduction (~ 70%) are based on the Hele-Shaw model. These results provide a useful reference for design, but their direct application to real dams should consider scale effects and site-specific conditions. The findings are valid for relative depths (d/H) = 0.026 to 0.165, and adjustments may be needed for different geometries or flow conditions.

**Fig. 8:**
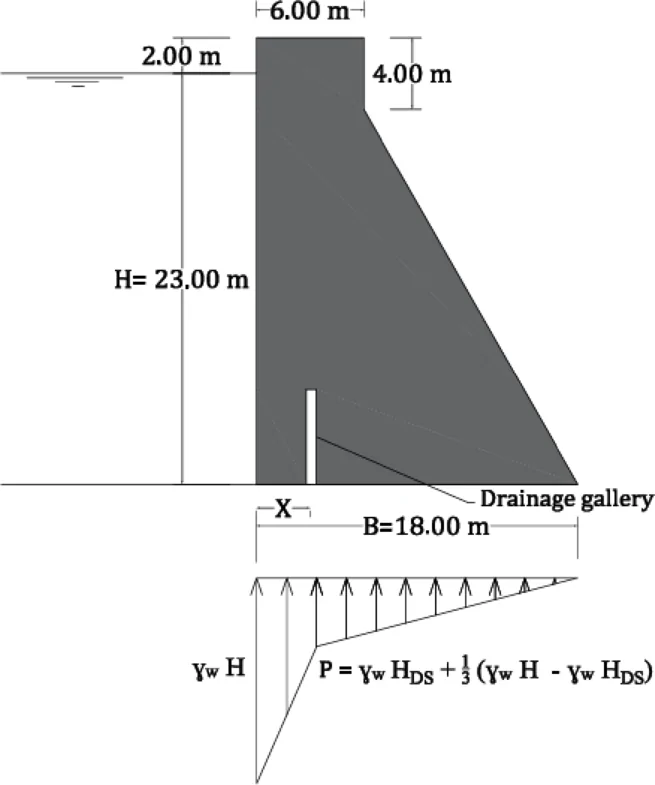
The assumed model dimensions and the theoretical uplift pressure distribution.

**Fig. 9:**
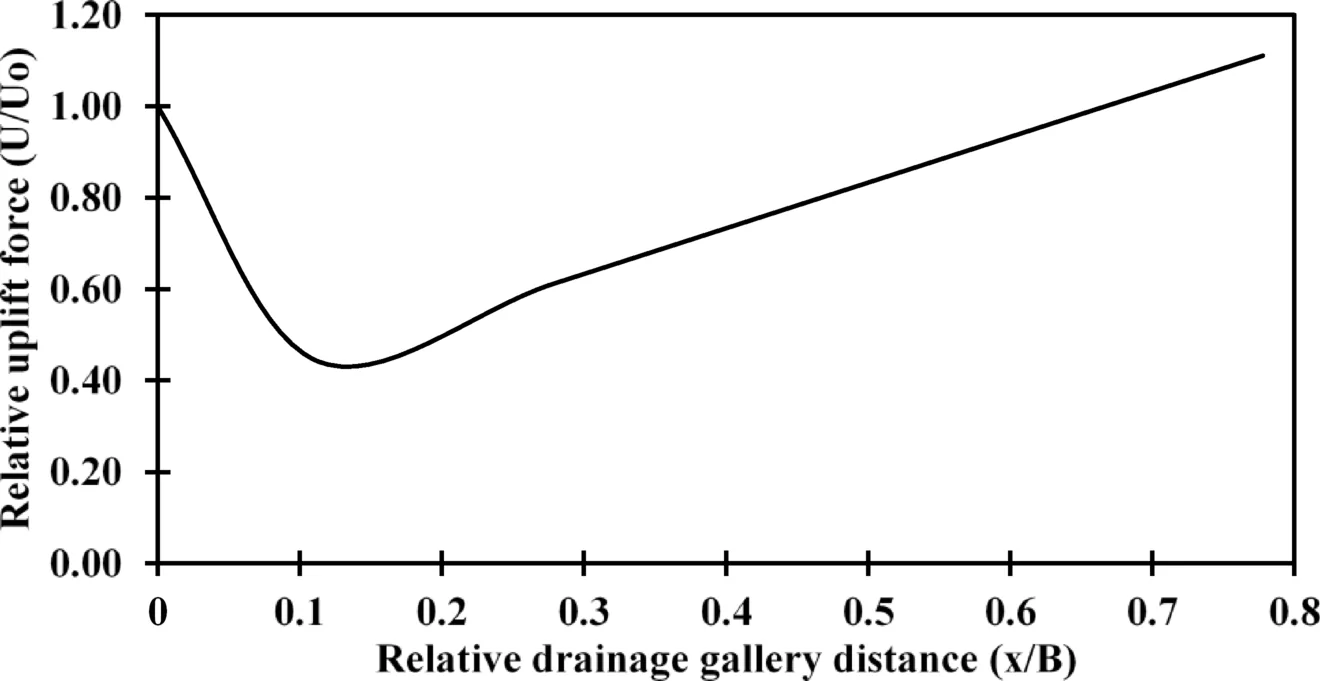
Relative uplift pressure force (U/U_o_) versus drainage gallery distance (x/B) for the theoretical analysis.

Theoretical calculations of the uplift force with and without a drainage gallery are performed on the assumed model shown in Fig. 8 and presented in Table 3. The assumed model is examined to find the optimum position of the drainage gallery. For the theoretical analysis, the uplift pressure at the drainage gallery position is according to USBR guidelines^11^.The assumed model is used to simulate the laboratory dam model and verify the experimental results. The laboratory model is measured in centimeters (cm), while the assumed model is measured in meters (m). Both models have the same geometric proportions, which allow a direct comparison between the experimental results and the theoretical calculations. This helps to check the accuracy of the laboratory results. The relationship between relative uplift force (U/U_o_) and drainage gallery distance (x/B) is shown in Fig. 9, which indicates that the minimum uplift force occurs at (x/B) = 0.125. The reduction percentage in this case seems to reach its maximum at (x/B) = 0.125, with (U.R.) = 56%.

**Table 3:** The theoretical uplift pressure force at different drainage gallery distances (x/B) for the assumed model.

(x/B)	Relative uplift pressure force (U/U_o_)	Uplift reduction (U.R.)
0	1.00	0.00
0.11	0.44	0.56
0.28	0.61	0.39
0.44	0.78	0.22
0.61	0.94	0.06
0.67	1.00	0.00
0.78	1.11	Non

Based on the above analysis of the experimental work, the optimum position of the drainage gallery is at a distance (x/B) = 0.15. Comparing the experimental results with the theoretical results shows that the drainage depth affects the uplift force in the experimental approach but not in the theoretical analysis. Furthermore, theoretical work shows that the uplift pressure force at a relative distance (x/B) = 0.67 is equal to that without a drainage gallery. Whereas in the experimental work, this condition is not achieved.

## Future work

Future work should explore several complementary paths to expand and apply the current findings. First, 3-D physical and numerical experiments are necessary to capture out-of-plane flow paths and dam geometries that Hele-Shaw (2-D) analogs cannot depict; such studies would quantify three-dimensional effects on optimal gallery placement and exit gradients. Second, transient reservoir loading (filling/emptying cycles and flood pulses) should be considered to assess time-dependent uplift and adaptive drainage performance. Third, the observed quantitative gap between the experimental optimum and the classical theoretical prediction form Eq. 1^11^ encourages the development of improved semi-analytical models that explicitly incorporate gallery depth and heterogeneous foundation properties. Fourth, the numerical simulation (SEEP/W) can be used and then compared the results with the experimental model results. Fifth, a field validation studies or prototype-scale experiments are suggested as a future direction. Lastly, modern machine learning (ML) techniques offer a promising approach for integrating laboratory, numerical, and field data^14,25^: supervised models (e.g., gradient boosting, random forests, or deep neural networks) can be trained on multi-physics simulation outputs and experimental data to forecast uplift and seepage across complex design spaces. Such ML-driven frameworks could eventually offer rapid decision support for dam engineers, enabling near-real-time evaluation and optimization of drainage retrofits and monitoring strategies.

## Conclusion

This study provided a systematic experimental assessment of drainage-gallery performance beneath gravity dams using a Hele-Shaw analog model, with particular focus on both horizontal positioning and vertical installation depth. The main conclusion can be summarized as follows:

- The drainage gallery achieves its most effective performance at a relative horizontal location *(x/B)=0.15* and a relative depth *(d/H)=0.165*, yielding an uplift-pressure reduction of nearly 70%.
- Comparison with the classical theoretical prediction from Eq. 1^11^, which identifies an optimum at *(x/B)=0.125* with an estimated 56% uplift pressure reduction.
- Increasing the drainage-gallery depth systematically reduces uplift pressure force and increases the seepage discharge.
- Increasing the horizontal distance from the heel increases the uplift pressure force and decreases the seepage discharge.
- If the gallery is placed underneath the dam base incorrectly, it may not reduce uplift pressure enough. So, it is necessary to find the most practical and cost-effective position of drainage galleries under concrete gravity dams.

## Supplementary Information

Below is the link to the electronic supplementary material.

Supplementary Material 1

## Data Availability

All data generated or analysed during this study are included in this published article and its supplementary information files].
