# Supplementary material for: An experimental study of the optimum position of the drainage gallery beneath concrete gravity dam
Source: Sci Rep. 2026 Aug 22;16:26360. doi: 10.1038/s41598-026-65760-6 (PMC13499808; doi:10.1038/s41598-026-65760-6)
Supplement: Supplementary file 1 [file 41598_2026_65760_MOESM1_ESM.docx]

**The data of the uplift pressure:**

**Table 1 The piezometers reading (in cm) for opened drains No. 1 to 5, (x/B = 0.11).**

| **Opened drains No. 1 to 5** | | | | | | | |
| --- | --- | --- | --- | --- | --- | --- | --- |
| **Piezometer No.** | | **I** | **II** | **III** | **IV** | **V** | **VI** |
| **Opened drains No.** | **(d/H_U.S._)** | **Piezometer reading in cm** | | | | | |
|  | **0.00** | **17** | **15** | **13.2** | **11.9** | **9.5** | **7** |
| **1** | **0.026** | 8.4 | 9 | 8.4 | 7.6 | 6.5 | 5 |
| **1, 2** | **0.061** | 5.9 | 6.7 | 6.6 | 6.1 | 5.3 | 4.3 |
| **1, 2, 3** | **0.096** | 4.5 | 4.9 | 5 | 4.9 | 4.3 | 3.5 |
| **1 to 4** | **0.130** | 3.6 | 3.7 | 3.8 | 3.9 | 3.5 | 3 |
| **1 to 5** | **0.165** | 3 | 2.6 | 2.8 | 3 | 2.6 | 2.4 |

**Table 2 The piezometers reading (in cm) for opened drains No. 6 to 10, (x/B = 0.28).**

| **Opened drains No. 6 to 10.** | | | | | | | |
| --- | --- | --- | --- | --- | --- | --- | --- |
| **Piezometer No.** | | **I** | **II** | **III** | **IV** | **V** | **VI** |
| **Opened drains No.** | **(d/H_U.S._)** | **Piezometer reading in cm** | | | | | |
|  | **0.00** | **17** | **15** | **13.2** | **11.9** | **9.5** | **7** |
| **6** | **0.026** | 12.9 | 7.6 | 7.7 | 7.3 | 6.4 | 5 |
| **6, 7** | **0.061** | 11.1 | 5.2 | 5.4 | 5.6 | 5 | 4 |
| **6, 7, 8** | **0.096** | 9.8 | 3.9 | 3.8 | 4.3 | 3.9 | 3.2 |
| **6 to 9** | **0.130** | 8.8 | 3.1 | 2.7 | 3.3 | 3.1 | 2.7 |
| **6 to 10** | **0.165** | 8.1 | 2.5 | 1.8 | 2.4 | 2.4 | 2.2 |

**Table 3** **The piezometers reading (in cm) for opened drains No. 11 to 15, (x/B = 0.44).**

| **Opened drains No. 11 to 15.** | | | | | | | | |
| --- | --- | --- | --- | --- | --- | --- | --- | --- |
| **Piezometer No.** | | **I** | **II** | | **III** | **IV** | **V** | **VI** |
| **Opened drains No.** | **(d/H_U.S._)** | **Piezometer reading in cm** | | | | | | |
|  | **0.00** | **17** | **15** | **13.2** | | **11.9** | **9.5** | **7** |
| **11** | **0.026** | 14.2 | 10.6 | | 6.2 | 6.7 | 6.1 | 4.8 |
| **11, 12** | **0.061** | 12.7 | 8.7 | | 4 | 4.8 | 4.5 | 3.8 |
| **11, 12, 13** | **0.096** | 11.9 | 7.6 | | 3 | 3.6 | 3.6 | 3.1 |
| **11, to 14** | **0.130** | 11.1 | 6.7 | | 2.2 | 2.6 | 2.7 | 2.5 |
| **11, to 15** | **0.165** | 10.4 | 5.9 | | 1.6 | 1.7 | 2 | 2 |

**Table 4** **The piezometers reading (in cm) for opened drains No. 16 to 20, (x/B = 0.61).**

| **Opened drains No. 16 to 20.** | | | | | | | |
| --- | --- | --- | --- | --- | --- | --- | --- |
| **Piezometer No.** | | **I** | **II** | **III** | **IV** | **V** | **VI** |
| **Opened drains No.** | **(d/H_U.S._)** | **Piezometer reading in cm** | | | | | |
|  | **0.00** | **17** | **15** | **13.2** | **11.9** | **9.5** | **7** |
| **16** | **0.026** | 15 | 12 | 9.1 | 5.4 | 5.7 | 4.7 |
| **16, 17** | **0.061** | 14 | 10.7 | 7.3 | 3.6 | 4.1 | 3.8 |
| **16, 17, 18** | **0.096** | 13.2 | 9.6 | 6.2 | 2.6 | 3 | 3 |
| **16, to 19** | **0.130** | 12.6 | 8.8 | 5.3 | 1.9 | 2.1 | 2.3 |
| **16, to 20** | **0.165** | 12 | 8.2 | 4.5 | 1.4 | 1.5 | 1.8 |

**Table 5 The piezometers reading (in cm) for opened drains No. 21 to 25, (x/B = 0.78).**

| **Opened drains No. 21 to 25.** | | | | | | | |
| --- | --- | --- | --- | --- | --- | --- | --- |
| **Piezometer No.** | | **I** | **II** | **III** | **IV** | **V** | **VI** |
| **Opened drains No.** | **(d/H_U.S._)** | **Piezometer reading in cm** | | | | | |
|  | **0.00** | **17** | **15** | **13.2** | **11.9** | **9.5** | **7** |
| **21** | **0.026** | 15.6 | 13 | 10.6 | 8.2 | 4.7 | 4.5 |
| **21, 22** | **0.061** | 15 | 12 | 9.4 | 6.8 | 3.2 | 3.5 |
| **21, 22, 23** | **0.096** | 14.5 | 11.3 | 8.5 | 5.8 | 2.3 | 2.5 |
| **21, to 24** | **0.130** | 13.9 | 10.6 | 7.7 | 5 | 1.6 | 1.8 |
| **21, to 25** | **0.165** | 13.4 | 10.1 | 7 | 4.2 | 1.1 | 1.3 |

**The data of the seepage quantity:**

**Table 6 The volume of the seeping oil** **for opened drains No. 1 to 5, (x/B = 0.11).**

| **Opened drains No. 1 to 5** | | | | |
| --- | --- | --- | --- | --- |
| **Opened drains No.** | **(d/H_U.S._)** | **Temp. (C)** | **Vol. (cm^3^)** | **Time (sec)** |
| **1** | **0.026** | 31 | 162.5 | 90.34 |
| **1, 2** | **0.061** | 31 | 235 | 90.56 |
| **1, 2, 3** | **0.096** | 31 | 285 | 90.5 |
| **1 to 4** | **0.130** | 31 | 327.5 | 90.03 |
| **1 to 5** | **0.165** | 31 | 365 | 90.32 |

**Table 7 The volume of the seeping oil** **for opened drains No. 6 to 10, (x/B = 0.28).**

| **Opened drains No. 6 to 10** | | | | |
| --- | --- | --- | --- | --- |
| **Opened drains No.** | **(d/H_U.S._)** | **Temp. (C)** | **Vol. (cm^3^)** | **Time (sec)** |
| **6** | **0.026** | 31 | 135 | 90.52 |
| **6, 7** | **0.061** | 31 | 190 | 90.29 |
| **6, 7, 8** | **0.096** | 31 | 240 | 90.35 |
| **6 to 9** | **0.130** | 31 | 272.5 | 90.36 |
| **6 to 10** | **0.165** | 31 | 300 | 90.34 |

**Table 8** **The volume of the seeping oil** **for opened drains No. 11 to 15, (x/B = 0.44).**

| **Opened drains No. 11 to 15** | | | | |
| --- | --- | --- | --- | --- |
| **Opened drains No.** | **(d/H_U.S._)** | **Temp. (C)** | **Vol. (cm^3^)** | **Time (sec)** |
| **11** | **0.026** | 34.5 | 117.5 | 90.43 |
| **11, 12** | **0.061** | 34.5 | 165 | 90.33 |
| **11, 12, 13** | **0.096** | 34.5 | 207.5 | 90.46 |
| **11, to 14** | **0.130** | 34.5 | 235 | 90.31 |
| **11, to 15** | **0.165** | 34.5 | 260 | 90.41 |

**Table 9** **The volume of the seeping oil** **for opened drains No. 16 to 20, (x/B = 0.61).**

| **Opened drains No. 16 to 20** | | | | |
| --- | --- | --- | --- | --- |
| **Opened drains No.** | **(d/H_U.S._)** | **Temp. (C)** | **Vol. (cm^3^)** | **Time (sec)** |
| **16** | **0.026** | 32.5 | 105 | 90.54 |
| **16, 17** | **0.061** | 32.5 | 150 | 90.34 |
| **16, 17, 18** | **0.096** | 32.5 | 182.5 | 90.22 |
| **16, to 19** | **0.130** | 32.5 | 230 | 90.38 |
| **16, to 20** | **0.165** | 32.5 | 255 | 90.4 |

**Table 10 The volume of the seeping oil** **for opened drains No. 21 to 25, (x/B = 0.78).**

| **Opened drains No. 21 to 25** | | | | |
| --- | --- | --- | --- | --- |
| **Opened drains No.** | **(d/H_U.S._)** | **Temp. (C)** | **Vol. (cm^3^)** | **Time (sec)** |
| **21** | **0.026** | 30.5 | 90 | 90.42 |
| **21, 22** | **0.061** | 30.5 | 130 | 90.38 |
| **21, 22, 23** | **0.096** | 30.5 | 160 | 90.5 |
| **21, to 24** | **0.130** | 30.5 | 185 | 90.39 |
| **21, to 25** | **0.165** | 30.5 | 210 | 90.27 |
